# Effects of electromagnetic fields treatment on rat critical-sized calvarial defects with a 3D-printed composite scaffold

**DOI:** 10.1186/s13287-020-01954-7

**Published:** 2020-10-06

**Authors:** Chang Tu, Jingyuan Chen, Chunwei Huang, Yifan Xiao, Xiangyu Tang, Hao Li, Yongzhuang Ma, Jiyuan Yan, Weigang Li, Hua Wu, Chaoxu Liu

**Affiliations:** 1 Department of Orthopedics, Renmin Hospital of Wuhan University, Wuhan, Hubei P.R. China; 2 Department of Orthopedics, Tongji Hospital, Tongji Medical College, Huazhong University of Science and Technology, Wuhan, Hubei P.R. China; 3 Department of Pathology and Pathophysiology, Medical College, Jianghan University, Wuhan, Hubei P.R. China; 4 Department of Radiology, Tongji Hospital, Tongji Medical College, Huazhong University of Science and Technology, Wuhan, Hubei P.R. China

**Keywords:** Electromagnetic fields, Mesenchymal stem cells, 3D-print, PLA/HA, Critical-sized defect

## Abstract

**Background:**

Current strategies for craniofacial defect are faced with unmet outcome. Combining 3D-printing with safe, noninvasive magnetic therapy could be a promising breakthrough.

**Methods:**

In this study, polylactic acid/hydroxyapatite (PLA/HA) composite scaffold was fabricated. After seeding rat bone marrow mesenchymal stem cells (BMSCs) on scaffolds, the effects of electromagnetic fields (EMF) on the proliferation and osteogenic differentiation capacity of BMSCs were investigated. Additionally, 6-mm critical-sized calvarial defect was created in rats. BMSC-laden scaffolds were implanted into the defects with or without EMF treatment.

**Results:**

Our results showed that PLA/HA composite scaffolds exhibited uniform porous structure, high porosity (~ 70%), suitable compression strength (31.18 ± 4.86 MPa), modulus of elasticity (10.12 ± 1.24 GPa), and excellent cyto-compatibility. The proliferation and osteogenic differentiation capacity of BMSCs cultured on the scaffolds were enhanced with EMF treatment. Mechanistically, EMF exposure functioned partly by activating mitogen-activated protein kinase (MAPK) or MAPK-associated ERK and JNK pathways. In vivo, significantly higher new bone formation and vascularization were observed in groups involving scaffold, BMSCs, and EMF treatment, compared to scaffold alone. Furthermore, after 12 weeks of implanting, craniums in groups including scaffold, BMSCs, and EMF exposure showed the greatest biomechanical properties.

**Conclusion:**

In conclusion, EMF treatment combined with 3D-printed scaffold has great potential applications in craniofacial regeneration.

## Background

Craniofacial defect caused by trauma, disease, congenital malformation, or surgery remains a challenge for surgeons [1, 2]. Autologous bone grafts, allografts, and xenografts are widely used for craniofacial defect regeneration [3, 4]. However, drawbacks including disease transmission, morbidity in donor site, lack of blood supply, and immune rejection limit their application [5]. Bone tissue engineering has emerged as a practical and promising solution. Especially recently, 3D-printing, as a rapidly evolving field, has showed its unique advantages. In contrast to traditional methods for fabricating 3D scaffolds, 3D-printing technique can provide customized implants with exact structure from computer-assisted design based on computerized tomography (CT) or magnetic resonance imaging (MRI) data files of patients [6, 7].

Polylactic acid (PLA) is a biodegradable polymer approved by the US Food and Drug Administration (FDA) for biomedical application [8, 9]. As a scaffold ingredient, it has been extensively applied in tissue engineering both in vitro and in vivo [10, 11]. However, shortcomings including brittle quality, inflammatory reactions limit its further application [12]. Hydroxyapatite (HA), as the major mineral constituent of the bone matrix, exhibits suitable mechanical properties. In contrast to PLA alone, combination of PLA and HA can improve the biocompatibility and the osteoconductivity of the scaffold [13]. It has been reported that scaffolds fabricated by different proportions of PLA and HA showed promising effects in bone tissue engineering [13–15]. Besides, incorporation of HA can markedly improve the hydroscopicity of PLA, thus promoting the swelling properties of scaffold. Furthermore, among various PLA to HA weight ratio for manufacturing implants by supercritical CO_2_, the PLA/HA (4:1) exhibited the optimal property [16].

Electromagnetic fields (EMF) have been used for the treatment of bone disorders including nonunion fractures, osteonecrosis and osteoporosis for many years [17, 18]. Various growth factors and proteins, as the vital component of tissue engineering, have been extensively investigated for application. However, due to the high cost and the fast clearance in vivo, the outcomes are far from satisfaction [19]. Replacing these stimulating factors with safe, noninvasive EMF therapy may open up new avenues.

Bone marrow mesenchymal stem cells (BMSCs) are the cornerstone in the fields of basic science and medicine due to excellent regenerative, reparative and angiogenic properties [20]. BMSCs can replicate as undifferentiated cells or commit to osteoblast, chondrocyte, tenocyte, endotheliocyte, adipocyte, and myocyte [21]. In bone tissue engineering, BMSCs are often chosen as the seeded cells. Moreover, it has been reported that undifferentiated BMSCs were more suitable for bone reconstruction compared to differentiated ones [22].

Critical-sized defect (CSD) model is often used by researchers to investigate fracture healing and corresponding therapies, as it impedes adequate blood supply of the fracture site or proper stability of the defect [23]. CSD models offer an environment where researcher can focus on the effects of the experimental strategies, excluding a variety of other interference factors. For adult rat, significant evidence indicates that defect over 5 mm in diameter could be considered a CSD [24].

Therefore, in this study, we combined BMSCs and 3D-printed composite scaffold to construct a calvarial graft. Sinusoidal EMF (15 HZ, 1 mT, 4 h/day) was selected as a stimulus. The effects of EMF treatment on seeded BMSCs proliferation and osteogenic differentiation capacity were explored in vitro. In vivo, constructed grafts were implanted to rat critical-sized calvarial defects and rats were treated with EMF. The bone formation as well as local vascularization was evaluated. We expected to explore a promising way for craniofacial regeneration.

## Methods

### Reagents

Polylactic acid (PLA) and nanograde hydroxyapatite (HA) were obtained from Sigma-Aldrich (St. Louis, MO, USA). Fetal bovine serum (FBS) was acquired from Gibco (NY, USA). Sinusoidal electromagnetic fields facility was described the same as in our previous study [25]. Dulbecco’s modified Eagle’s medium F12 (DMEM/F12) was procured from Hyclone (Logan, UT, USA). Osteoinductive medium (OIM), chondroinductive medium (CIM), and adipoinductive medium (AIM) were obtained from Cyagen Biosciences (USA). For western blot analysis, antibodies against RUNX2 and OPN were obtained from Abcam (Cambridge, MA, USA). Antibodies specific for p-ERK, ERK1/2, p-JNK, JNK, p-p38, p38 were purchased from Cell Signaling Technology (Beverly, MA, USA). Antibodies against GAPDH and secondary antibodies were purchased from Boster (Wuhan, China). For flow cytometry analysis, antibodies specific for FITC-CD44, PE-CD90, and corresponding isotypes were procured from BD Biosciences (New Jersey, USA).

### Rat BMSCs isolation and identification

Six to eight-week-old Sprague-Dawley (SD) rats (male, 60–100 g) were procured from the Laboratory Animal Center of Tongji Hospital of Hubei province in China. BMSCs were isolated as described before [25]. Concisely, BMSCs were obtained by flushing bone marrow out of the femurs and tibias of rats using growth medium (GM) including DMEM/F12 supplemented with 10% fetal bovine serum (FBS; Gibco, NY, USA), 100 units/ml penicillin, and 100 units/ml streptomycin (Sigma-Aldrich). Cells were cultured at 37 °C in 5% CO_2_ and nonadherent cells were removed during every passage. Passage 3 was used for subsequent experiments.

For BMSCs identification, the BMSCs-multipotent potential for differentiation toward adipogenic, osteogenic, and chondrogenic lineages and surface markers were detected. Briefly, cells were cultured with AIM, OIM, and CIM, respectively, following the protocol from Cyagen Biosciences. Subsequently, cells or sections of cell pellet were stained with corresponding Oil Red O, Alizarin Red S, and Alcian Blue (Sigma-Aldrich). In addition, the percentage of CD40^+^CD90^+^ BMSCs was confirmed by flow cytometry (BD LRII, BD Biosciences).

### Fabrication of PLA/HA composite scaffolds by 3D-printing

**Fig. 1:**
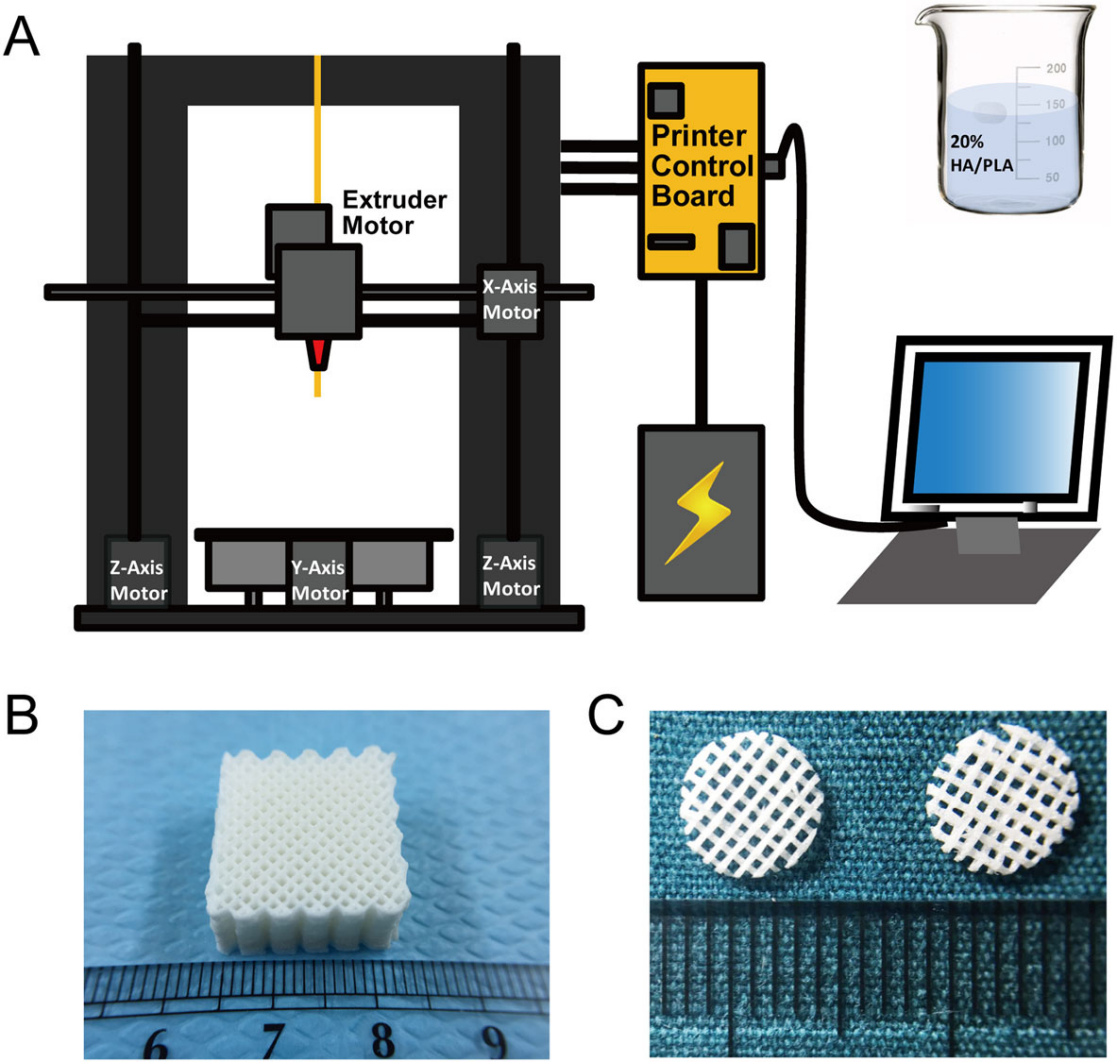
Exhibition of the nozzle-deposition system. **a** 3D printing device for fabricating PLA/HA composite scaffolds. **b** Gross observation of the cubic scaffold, each unit of the scale indicates 1 mm. **c** Gross observation of the cylindrical scaffold, each unit of the scale indicates 1 mm

PLA and HA were mixed with a weight ratio of 4/1 and dissolved in dichloromethane (Sigma-Aldrich) using magnetic stirring. Then, the raw materials were processed to produce PLA/HA composite scaffolds using the nozzle-deposition system (Fig. 1) as previously reported [26]. During the process of 3D-printing, the parameters were as follows: the nozzles used for printing were 0.41 mm in diameter; the layer thickness of each print was 0.2 mm; the extrusion speed was set as constant 0.1 mm/s; the pore diameter was 1 mm. Macroporous cubic (1.8 cm length, 1.8 cm width, 1 cm height) or cylindrical (6-mm diameter, 0.6-mm height) scaffolds were constructed and dehydrated for further research. For cell or animal experiments, the 3D-printed scaffolds were sterilized with ethylene oxide before use.

### Characterization of 3D-printed scaffolds

For porosity evaluation, the 3D-printed scaffolds were measured as described before [27]. Briefly, apparent volume (*V*_a_) and dry weight (*W*_d_) of the cubic scaffold (*n* = 6) were measured. Subsequently, scaffolds were immersed in 95% ethanol for 5 min, washed with distilled water, and kept in distilled water over night. Then, the wet weight (*W*_w_) of the scaffolds was determined. ρ indicated the density of the distilled water. The scaffold porosity was calculated as:

$$ \mathrm{Porosity}\ \left(\%\right)=\frac{W_{\mathrm{w}}-{W}_{\mathrm{d}}}{{\uprho V}_{\mathrm{a}}}\times 100\% $$

For the mechanical properties of scaffolds, an Instron 5566 device (Instron Corporation, USA) was employed. Concisely, cubic specimens (*n* = 6) were placed vertically between two solid platens. The compression rate was set as 1 mm/min with a 5-N load cell. Compression strength was obtained according to the peak of the stress-strain curve. Modulus of elasticity was acquired from the initial linear portion of the stress-strain curve.

For scaffolds morphology evaluation, a scanning electron microscopy (SEM, TESCAN VEGA 3 LMU, CZ) was used following the manufacturer’s instructions. Briefly, cubic scaffolds with or without cells laden were fixed in 2.5% glutaraldehyde (PH = 7.4) for 24 h, and then dehydrated in a graded ethanol. Subsequently, samples were critical-dried and coated with gold. Finally, scaffolds were examined using SEM in different cross sections and magnifications.

### Cell viability and proliferation of the BMSCs cultured on the PLA/HA composite scaffolds

Cell viability and proliferation on the scaffolds was assessed by the CCK-8 assay (Boster) and a LIVE/DEAD kit (Sigma-Aldrich). Briefly, a suspension of 1 × 10^4^ BMSCs in 100 μl GM were loaded onto the cubic PLA/HA composite scaffolds. After 1, 3, 5, and 7 days of culturing, viability and proliferation of BMSCs were visualized using LIVE/DEAD kit. Live cells per microscopic field were counted using Image-J software. Furthermore, CCK-8 kit was employed to quantitatively evaluate the cell viability. The optical density (OD) value was read by a microplate reader at 450 nm (Bio Tek Instruments, Winooski, VT, USA).

### EMF on the proliferation of BMSCs cultured on the scaffolds

Rat BMSCs of passage 3 were seeded at a density of 1 × 10^4^ cells per cubic scaffold and cultured in GM. BMSCs were divided into two groups: GM group (without any treatment) and GM+EMF group (cells were treated 4 h/d with 1 mT, 15 Hz EMF). BMSCs were cultured on the scaffolds for 1, 3, 5, 7, and 9 days. Subsequently, CCk-8 kit was used to assess the cell viability at each time point.

To unearth the underlying mechanisms of EMF on the proliferation of BMSCs cultured on scaffolds, seeded BMSCs were culture in serum-free GM for 8 h and then treated with or without EMF for 30 min. Subsequently, western blotting was used to detect the phosphorylation of MAPK pathway. Briefly, cells were washed with phosphate buffered saline (PBS) and lysed with RIPA supplemented with 1% protease inhibitor cocktail and 1% phosphatase inhibitor cocktail (Boster). Then, 25 μg protein samples were electrophoretically separated on SDS-polyacrylamide gels and transferred to PVDF membrane using a Bio-rad blotting system. Subsequently, PVDF membranes were blocked with 5% bone serum albumin for 1 h at room temperature and incubated with corresponding antibodies overnight at 4 °C. Afterward, membranes were washed with TBST and incubated again with appropriate secondary antibodies for 1 h at room temperature. Finally, the bands were detected using enhanced ECL system (Thermo, USA). GAPDH was employed as the loading control and representative images were shown.

### EMF on the osteogenic differentiation potential of BMSCs cultured on the scaffolds

1 × 10^4^ rat BMSCs of passage 3 were seeded on the cubic scaffolds and cultured in GM. The culture medium was changed to OIM after the cell density reached 60%. Then, cells were cultured in OIM for further 7 days. BMSCs were divided into two groups: OIM group (cells were only cultured in OIM) and OIM+EMF group (cells were cultured in OIM and treated 4 h/d with 1 mT, 15 Hz EMF). Western blotting was employed to detect the osteogenesis-related protein.

To detect the underlying mechanisms of EMF on the osteogenic differentiation potential of BMSCs cultured on scaffolds, seeded cells were cultured in serum-free OIM for 8 h and then treated with or without EMF (1 mT, 15 HZ) for 30 min. Afterward, western blotting was used to evaluate the activation of MAPK pathway.

### Experiment design of rat critical-sized calvarial defects

One hundred and twenty-six 12–13-week-old male SD rats (280-320 g weight) were obtained from the Laboratory Animal Center of Tongji Hospital and were approved by the Committee. The experimental procedure was performed as described previously [28]. Briefly, animals were anesthetized by intraperitoneal injection of pentobarbital (3.5 mg/100 g weight) and inhaled isoflurane. A 1.5-cm incision down to the periosteum was made over the proximal-medial area of the scalp. Then, the periosteum was sharply divided in the middle and gently pushed laterally to expose the calvaria. All efforts were made to minimize the periosteal injury. Subsequently, a critical-sized defect (diameter = 6 mm) was created in the center of the skull using a micro-trephine. The uniform defects were randomly allocated to five groups: (1) control group without implant, control (*n* = 24); (2) acellular cylindrical 3D-printed composite scaffold, scaffold (*n* = 24); (3) cylindrical scaffold combined EMF treatment, scaffold/EMF (*n* = 24); (4) cylindrical scaffold seeded with BMSCs, scaffold/BMSCs (*n* = 24); and (5) BMSC-laden cylindrical scaffold combined EMF, scaffold/BMSCs/EMF (*n* = 24). After operation, the incision was sutured layer by layer. All animals survived after surgery and fully recovered after 24 h.

### Bone regeneration assessment by micro-CT

Six rats from each group were sacrificed using an overdose of anesthetics at 4 and 12 weeks. The harvested craniums were fixed in 10% paraformaldehyde for 2 days and then scanned by micro-CT (vivaCT 40, Scanco 274 Medical, Switzerland). All 3D images were reconstructed by VGStudio software with a constant threshold. Accordingly, the bone volume relative to total volume (BV/TV) and the bone mineral density (BMD) within the defect area were calculated (*n* = 6). After scanning, all samples were decalcified in 10% EDTA (PH 7.0) for 1 month and prepared for histological analysis.

### Neovascularization evaluation through micro-CT-based micro-angiography

For neovascularization evaluation, six rats from each group were examined using micro-angiography 6 weeks post-surgery. The vascular perfusion was performed using MICROFIL compounds (MV-122, Flow Tech, Carver, MA, USA) via the cardiac approach as mentioned before [29]. Concisely, the rat was anesthetized by intraperitoneal injection of pentobarbital and affixed to the fixation board. Then, the chest wall was opened to expose the heart. Afterward, a butterfly needle was inserted into the left ventricle, and the inferior vena cave was dissected. The perfusion fluid entered from the left ventricle and exited from the inferior vena cava. Accordingly, the vasculature was cleared with heparinized saline, fixed by 4% formaldehyde and infused with MICROFIL compounds. After polymerization, the samples were decalcified and scanned using micro-CT. VGStudio software was employed to evaluate the blood vessel area and blood vessel number in the calvarial defect.

### Histological evaluation

After CT scanning and decalcification, samples were harvested and dehydrated in ascending concentrations of alcohol. Then, craniums were embedded in paraffin and coronally cut into 5-μm-thick sections. Finally, the slices were stained with hematoxylin and esoin (HE) and Masson’s trichrome. New bone area fraction calculated as new bone area/defect area within the defect of each section was obtained using Image-J software (*n* = 6).

### Biomechanical analysis

At 12 weeks post-surgery, six craniums from each group were harvested for biomechanical analysis. The craniums of six normal 6-month-old rats served as the normal control. Push-out test was conducted to evaluate the mechanical properties of the craniums using a 6-mm-diameter push-out jig. Briefly, the jig was centered in the defect site of the craniums and pushed at a constant speed of 1 mm/min. The process was controlled and recorded using an Instron 5566 device (Instron Corporation, Norwood, MA, USA). Ultimate force (*F*) of each sample was obtained and ultimate stress (*σ*) was calculated accordingly.

### Statistical analysis

Values were shown as mean ± standard deviations. Statistical comparisons were assessed by Student’s *t* test or one-way ANOVA followed by Tukey post hoc test. Statistically significant was considered *P* < 0.05.

## Results

### Characterization of rat BMSCs

**Fig. 2:**
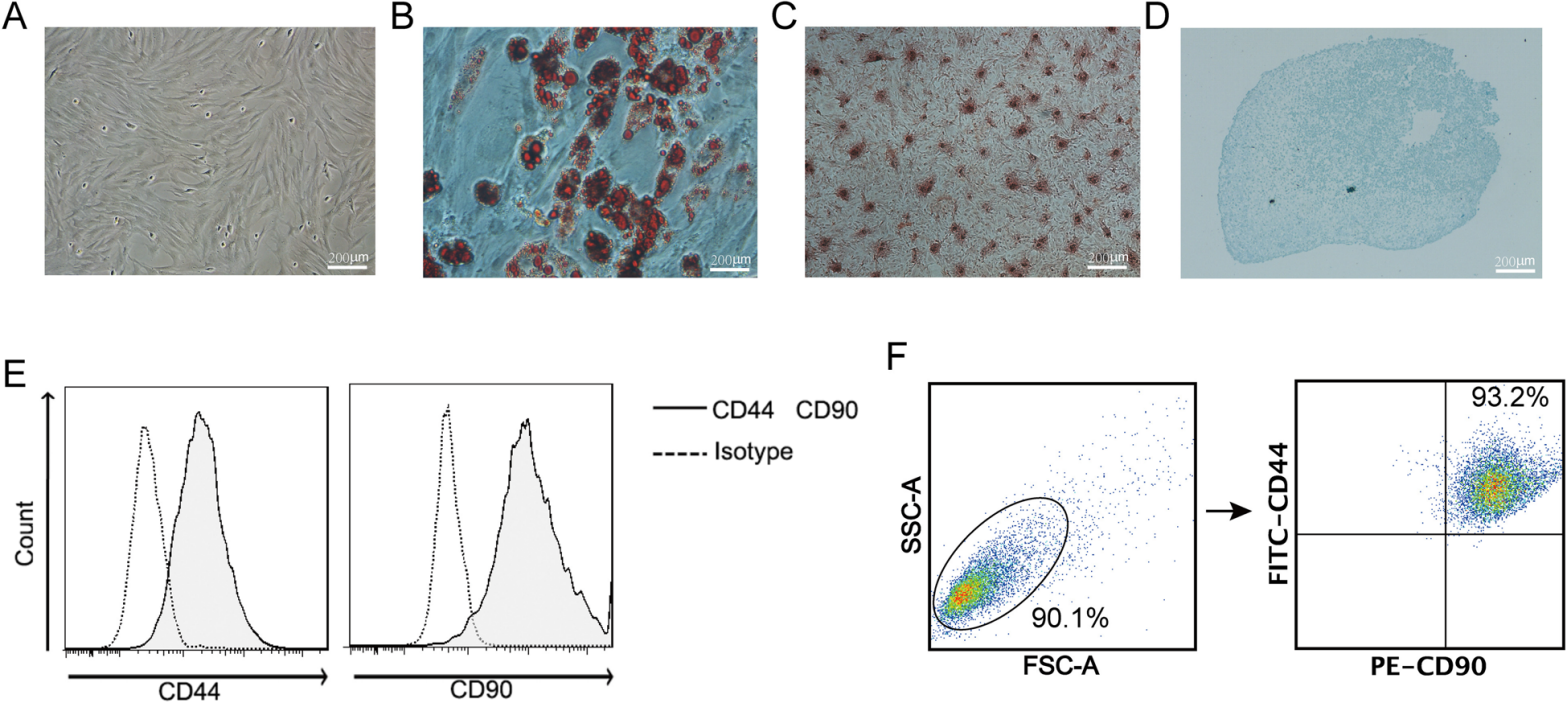
Characterization of rat BMSCs. **a** Cultured BMSCs exhibited a long spindled morphology by passage 3. **b** Intracellular oil droplets positively stained with Oil Red O. **c** Calcium nodules positively stained by Alizarin Red S. **d** Sections of cell pellet positively stained with Alcian Blue. **e** Biomarkers for BMSCs, CD44, and CD90, were used to identify the purity of BMSCs. Meanwhile, isotype antibodies for CD44 and CD90 were used as negative controls. **f** CD44^+^CD90^+^ cells were considered BMSCs

Cultured BMSCs exhibited a homogenous long spindle-shaped morphology by passage 3 (Fig. 2a). Oil Red O (Fig. 2b), Alizarin Red S (Fig. 2c), and Alcian Blue (Fig. 2d) staining confirmed the cells multipotent capacity for differentiation. 93.2% percentage of cells was positive stained with CD44 and CD90 (Fig. 2e, f).

### Characterization of 3D-printed PLA/HA composite scaffolds

**Fig. 3:**
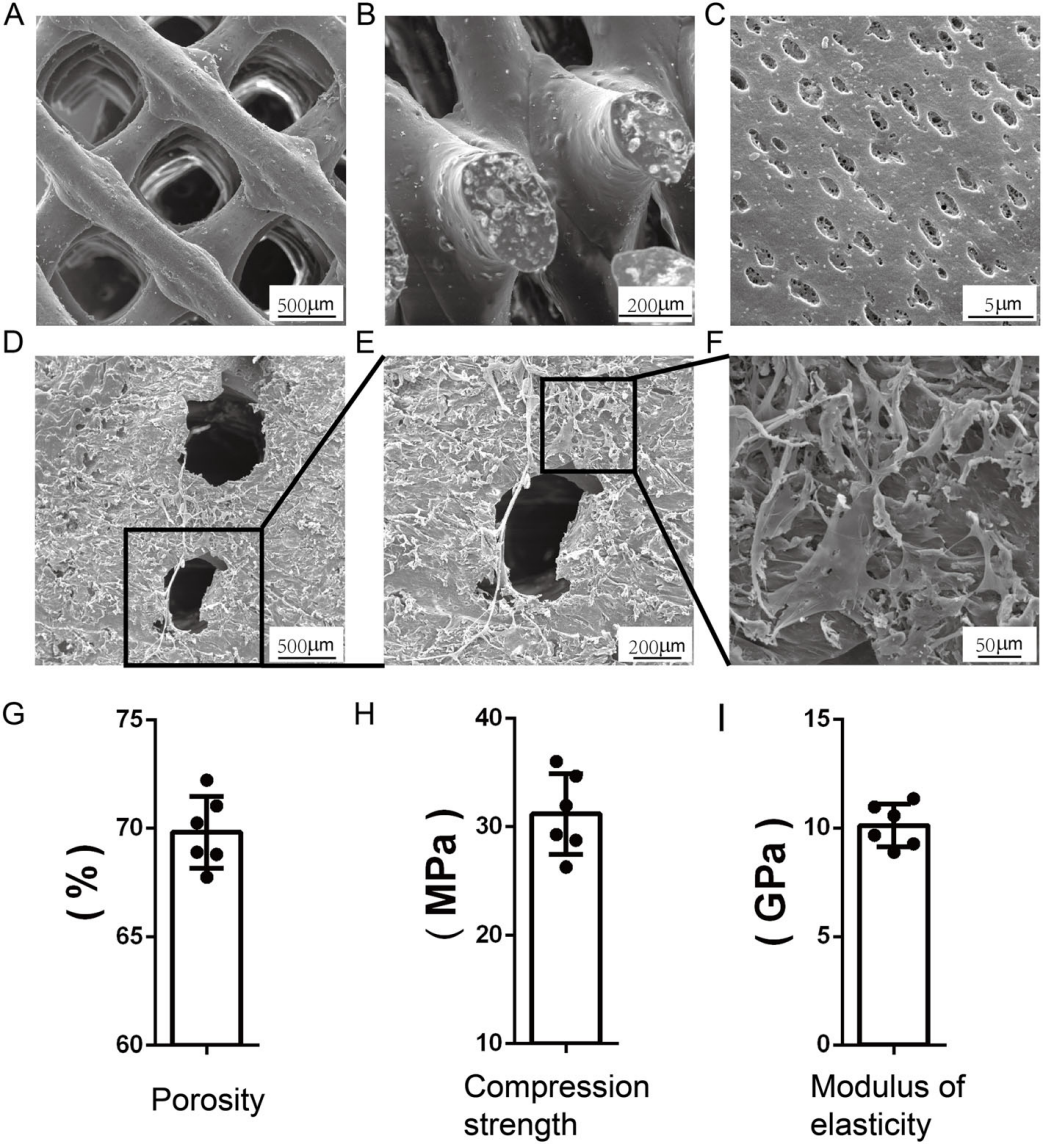
Typical SEM images of PLA/HA composite scaffolds morphology. **a** Surface of the blank scaffolds at low magnification. **b** Longitudinal section of the blank scaffolds at low magnification. **c** Surface of the blank scaffolds at high magnification. **d** Surface of the BMSC-laden scaffolds at low magnification. **e**, **f** Enlarged images. **g** Porosity of the scaffolds. **h** Compression strength of the scaffolds. **i** Modulus of elasticity of the scaffolds

In our study, the 3D-printed PLA/HA composite scaffolds showed an interconnected network of macropores with a porosity of 70 ± 2.23% (Fig. 3g). Stress-strain curves suggested that the fabricated scaffolds owned a compression strength of 31.18 ± 4.86 MPa (Fig. 3h) and a modulus elasticity of 10.12 ± 1.24GPa (Fig. 3i). SEM was applied to observe the morphology of the scaffolds with or without BMSCs. The surface (Fig. 3a) and longitudinal section (Fig. 3b) of the scaffold exhibited uniform interconnected and macroporous structure at low magnification. At high magnification, due to the dichloromethane volatilization in scaffolds, random small pores were observed on the surface (Fig. 3c). Seeded BMSCs appeared flat and fully spreaded out on the scaffolds (Fig. 3d, e, f).

### Cell viability and proliferation on the PLA/HA composite scaffolds

**Fig. 4:**
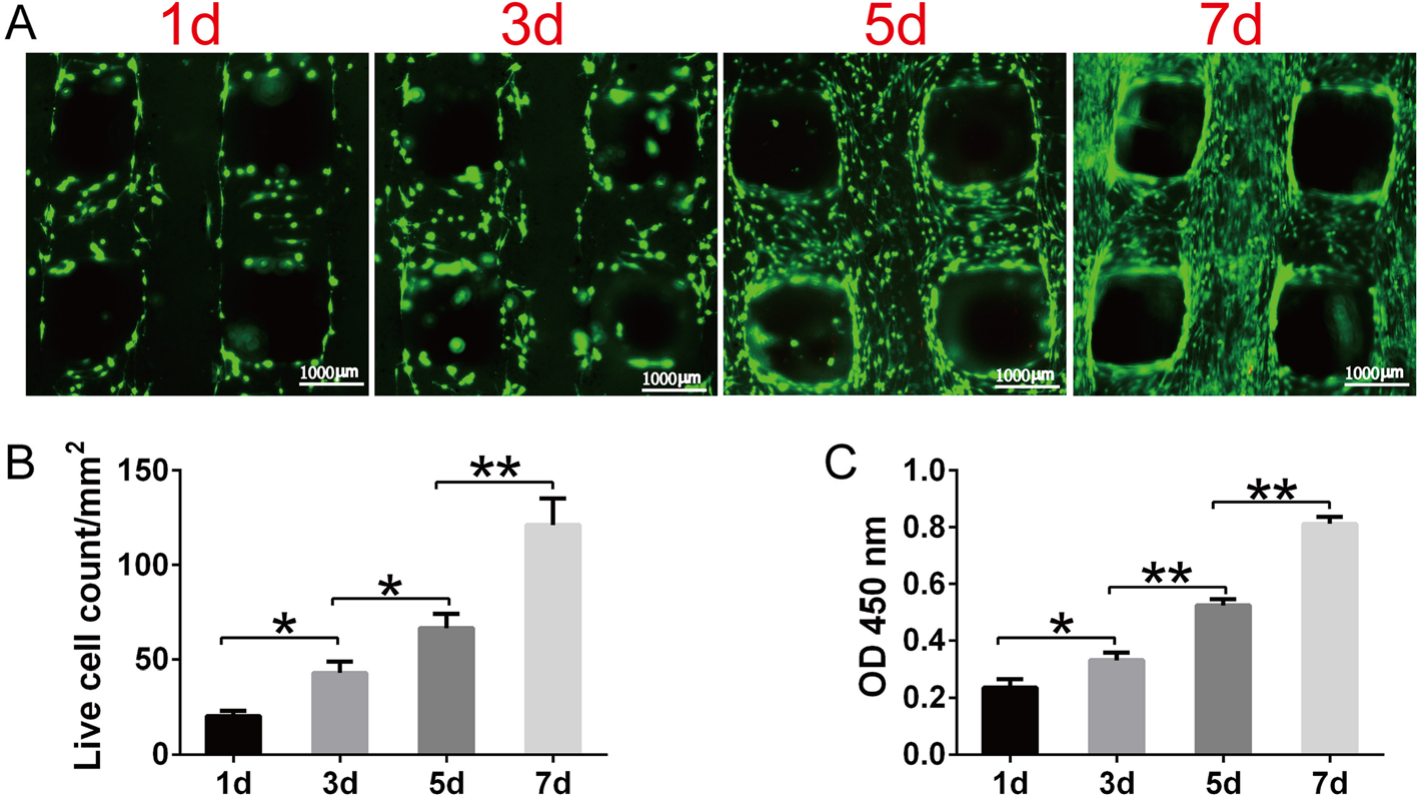
BMSC viability on the PLA/HA composite scaffolds. **a** LIVE/DEAD assay showing cell viability after 1, 3, 5, and 7 days culturing on scaffolds (dead cells, red; live cells, green). **b** Live cell density of BMSCs cultured on PLA/HA composite scaffolds at 1, 3, 5, and 7 days (*n* = 3). **c** CCK-8 assay showing cell proliferation after 1, 3, 5, and 7 days culturing on scaffolds (*n* = 3) (^*^P < 0.05, ^**^P < 0.01)

As shown by the LIVE/DEAD staining (Fig. 4a), seeded BMSCs increased over time. Furthermore, the quantification analysis of live cell (Fig. 4b) demonstrated a significantly higher numbers of living cells during the culture. Cell viability of the BMSCs cultured on scaffolds was confirmed by CCK-8 assay. Notable higher OD value was observed with culture time (Fig. 4c).

### Effects of EMF on the proliferation of BMSCs cultured on scaffolds

**Fig. 5:**
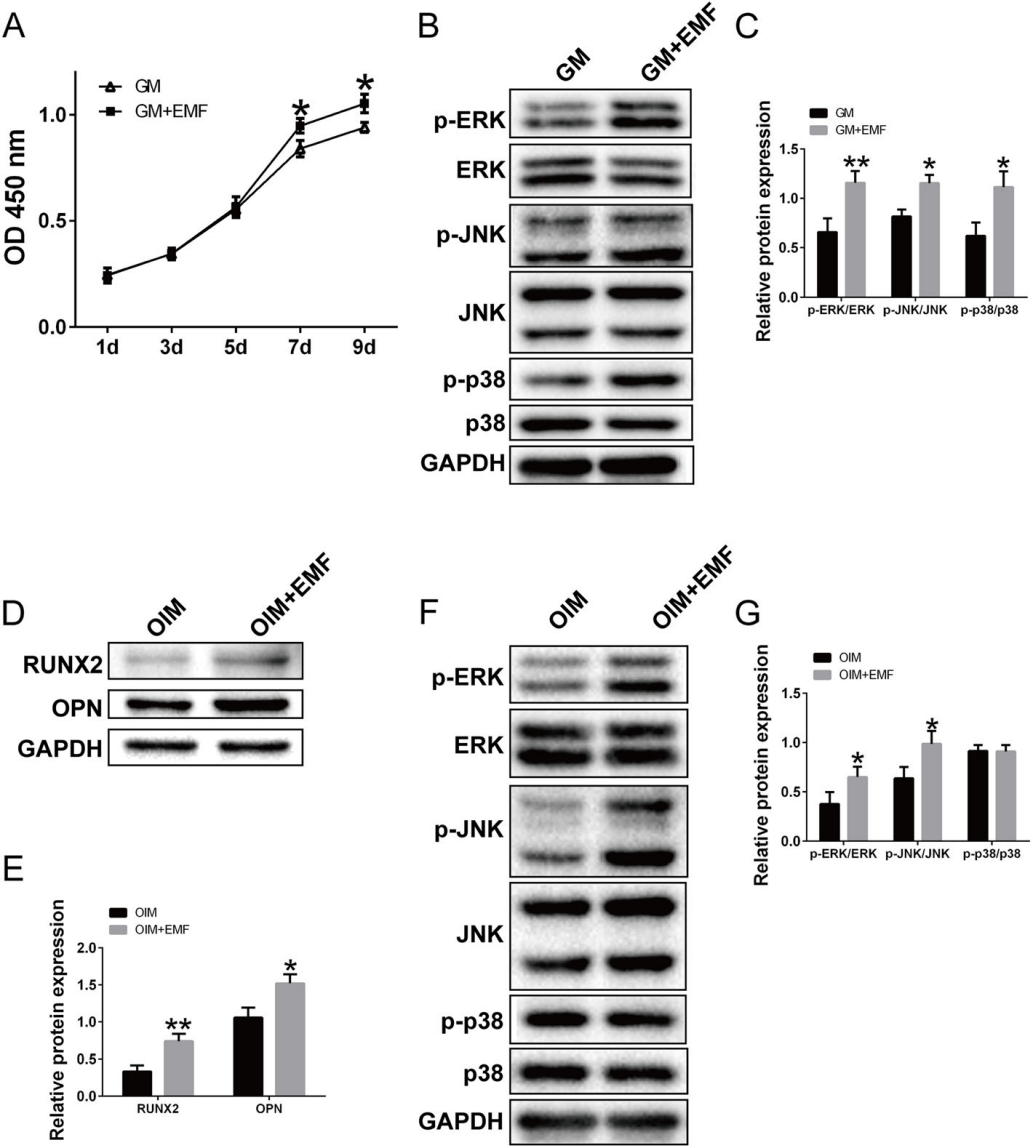
Effects of EMF on the proliferation and osteogenic differentiation of BMSCs cultured on scaffolds. **a** BMSCs were seeded on cubic scaffolds, cultured in GM, and treated with or without EMF. Proliferation of both groups was detected at 1, 3, 5, 7, and 9 days. **b** Phosphorylations of MAPK pathway involved in GM and GM+EMF groups assessed by western blotting. **c** Quantification analysis of the MAPK pathway activation in GM and GM+EMF groups using Image-J software, total ERK, JNK, and p38 were served as the internal control (*n* = 3). **d** BMSCs were seeded on cubic scaffolds, cultured in OIM, and treated with or without EMF for 7 days. Protein expression of RUNX2 and OPN of both groups were determined by western blotting. **e** Quantification analysis of the RUNX2 and OPN expression by Image-J software, GAPDH was used as the internal control (*n* = 3). **f** Activation of MAPK pathway involved in OIM and OIM+EMF groups evaluated by western blotting. **g** Quantification analysis of the MAPK pathway activation in OIM and OIM+EMF groups using Image-J software, total ERK, JNK, and p38 were used as the internal control (*n* = 3) (^*^P < 0.05, ^**^P < 0.01)

BMSCs were seeded on the scaffolds, cultured in GM, and treated with or without EMF for 1, 3, 5, 7, and 9 days. CCK-8 kit was conducted to evaluate the cell viability of the two groups. As shown in Fig. 5a, seeded BMSCs exhibited a higher proliferation level at day 7 and day 9. MAPK signal pathway is highly associated with the processes of proliferation and differentiation. Seeded BMSCs were cultured in serum-free GM for 8 h and treated with or without EMF for 30 min. As indicated in Fig. 5b and c, EMF exposure significantly promoted the phosphorylation of ERK, JNK and p38.

### Effects of EMF on the osteogenic differentiation capacity of BMSCs cultured on scaffolds

Cells were seeded on the scaffolds, cultured in OIM, and treated with or without EMF for 7 days. Western blotting was used to evaluate the osteogenesis-related protein expression of the two groups. As shown in Fig. 5d and e, the levels of RUNX2 and OPN were elevated after EMF treatment. Furthermore, seeded BMSCs were cultured in serum-free OIM for 8 h and treated with or without EMF for 30 min. As indicated in Fig. 5f and g, EMF treatment remarkably activated the phosphorylation of ERK and JNK.

### Construction of animal models and micro-CT evaluation for bone regeneration

**Fig. 6:**
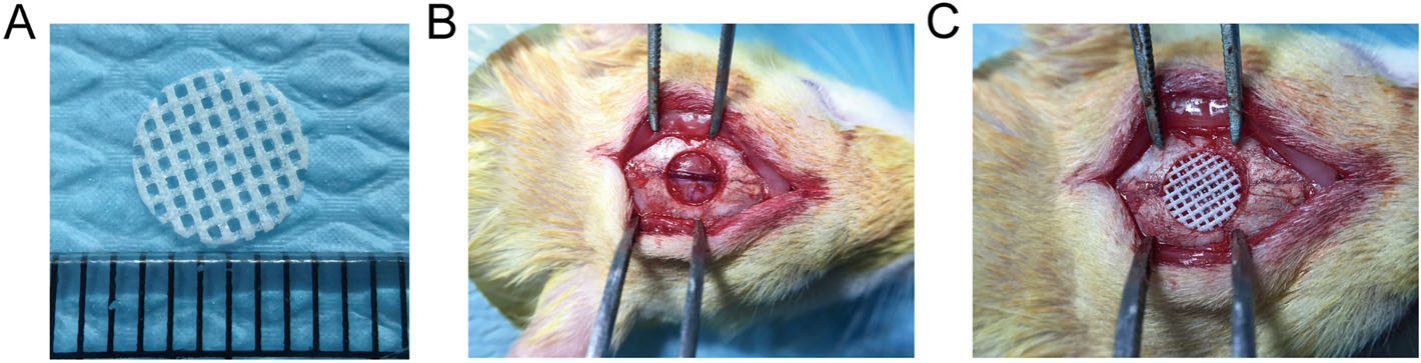
Construction of rat critical-sized calvarial defect. **a** Macroscopic view of the PLA/HA composite scaffold for implantation. **b** A 6-mm-diameter defect was created in the center of the skull using a micro-trephine. **c** Defect was implanted with the scaffold

**Fig. 7:**
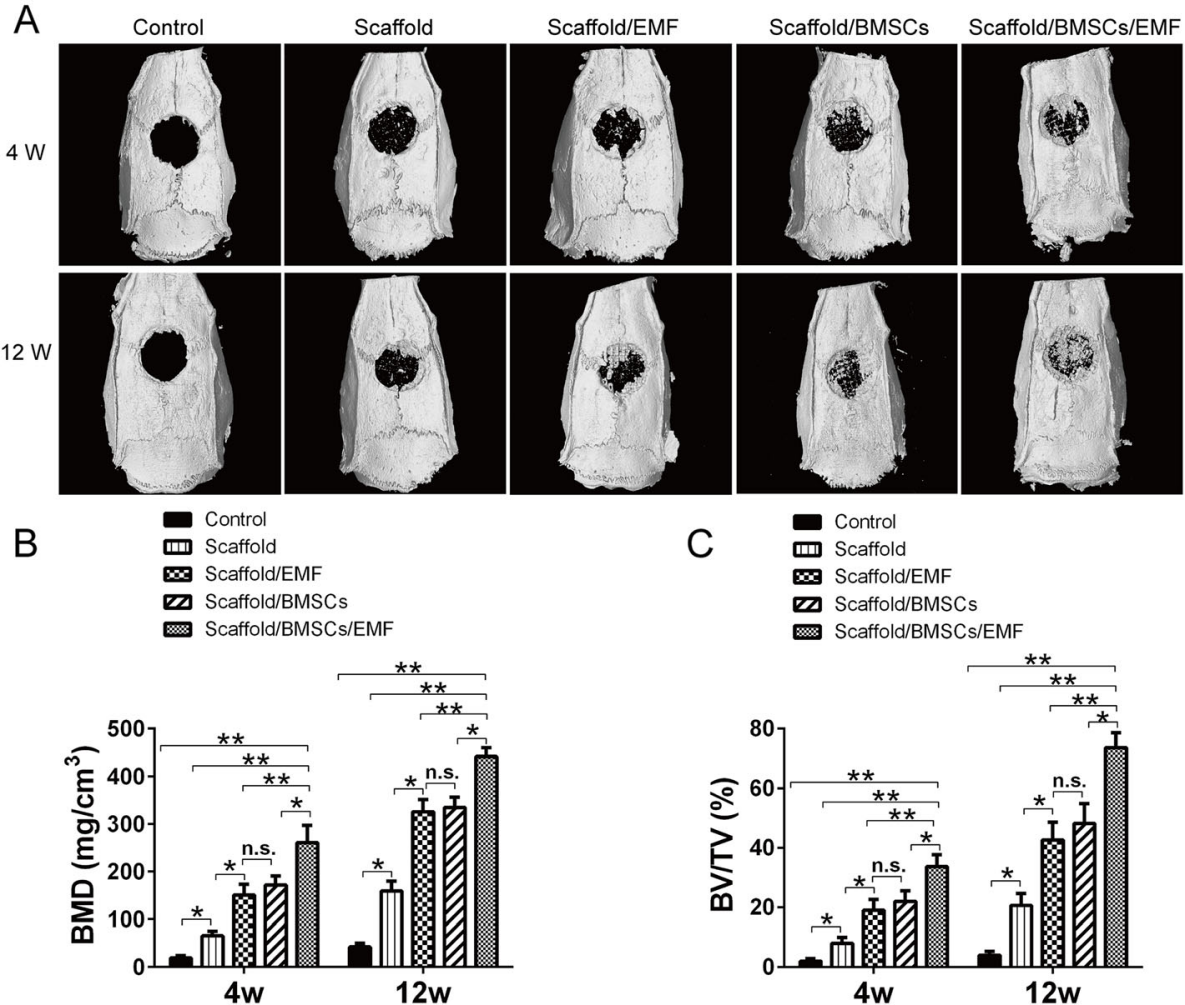
Bone regeneration evaluated by mico-CT. **a** Representative reconstructed mico-CT images of craniums in different groups at 4 and 12 weeks. **b** BV/TV and **c** BMD quantification analysis in every group at each time set (*n* = 6). n.s. indicated no significance (^*^P < 0.05, ^**^P < 0.01)

To evaluate the efficacy of EMF therapy combined with PLA/HA composite scaffolds in vivo, a 6-mm rat critical-sized calvarial defect model was constructed (Fig. 6). Morphology of the newly formed bone was assessed by micro-CT. Representative 3D reconstructed craniums of each group at 4 and 12 weeks were shown in Fig. 7a. Accordingly, little bone formation was observed in the control group. Groups including scaffold, BMSCs, and EMF exposure showed the greatest newly formed bone area at 4 and 12 weeks respectively. Furthermore, significant higher values of BV/TV and BMD were observed in scaffold/BMSCs/EMF group compared to other 4 groups at 4 and 12 weeks (Fig. 7b, c).

### Evaluation of neovascularization using micro-CT-based angiography

**Fig. 8:**
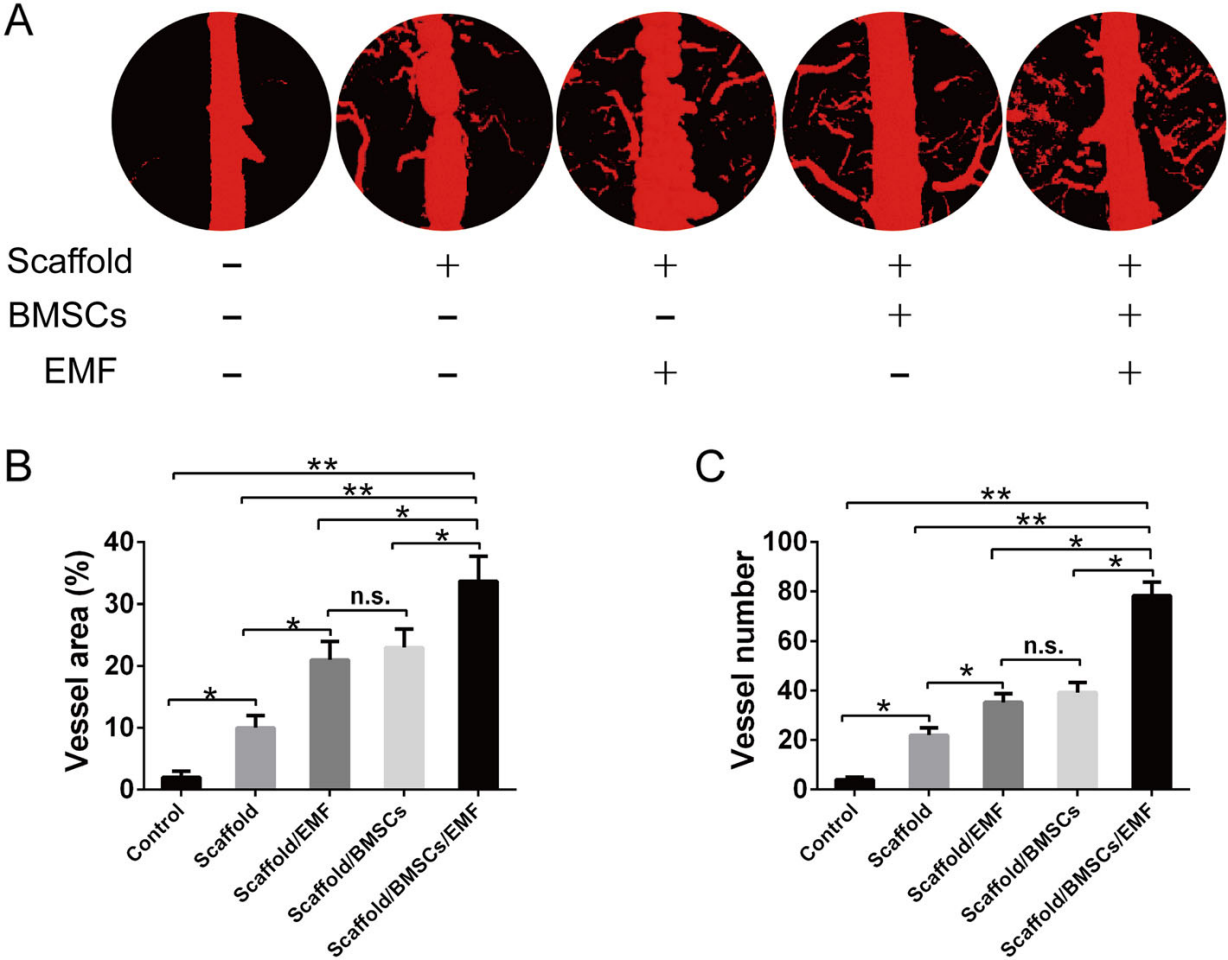
Neovascularization evaluated by micro-CT-based angiography. **a** Representative 3D reconstructed images of new blood vessels formation in the calvarial defects in each group at 6 weeks. **b** Local vessel area and **c** vessel number quantification analysis in different groups at 6 weeks (*n* = 6). n.s. indicated no significance (^*^P < 0.05, ^**^P < 0.01)

Six weeks after operation, vascularization of the bone defect area was evaluated by perfusing the vessels with MICROFIL compounds and imaging with micro-CT. As shown in Fig. 8a, higher formation of blood vessels and communicating branches were observed in scaffold/BMSCs/EMF group compared to other 4 groups at 6 weeks. Quantitative analysis of newly formed vessels was further performed using VGStudio software. As exhibited in Fig. 8b and c, little vessels formation was observed in control group. The vessel area and blood vessel number in scaffold/BMSCs/EMF group showed the highest value among the five groups.

### Histological evaluation of bone formation in the calvarial defect

**Fig. 9:**
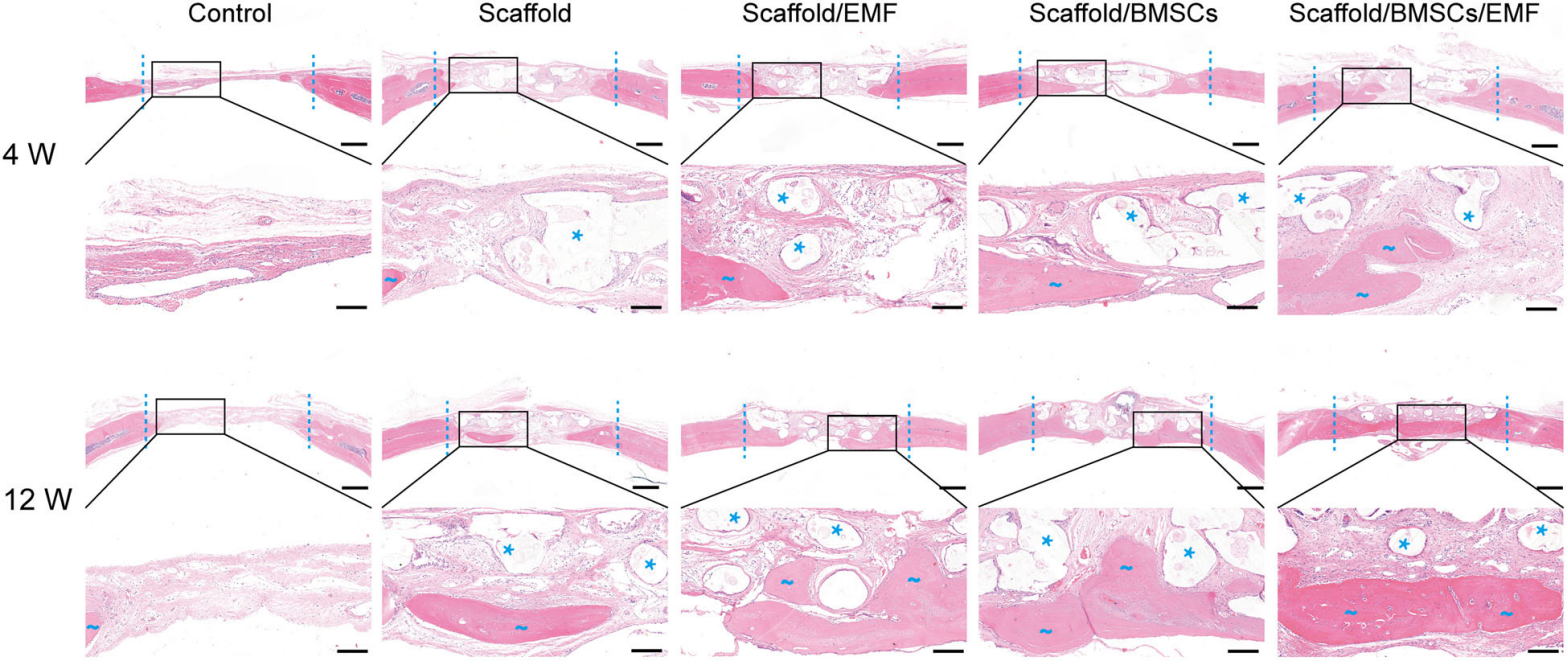
Bone regeneration evaluated by HE staining. Coronal HE stained sections in the calvarial defect region of different groups were taken 4 and 12 weeks post-operation. The dotted line indicates the boundary of the 6-mm defect. Blue wary lines designate newly formed bone, and asterisks point to residual scaffolds. Scale bars in lower magnification images represent 1000 μm, and scale bars in higher magnification images represent 250 μm

**Fig. 10:**
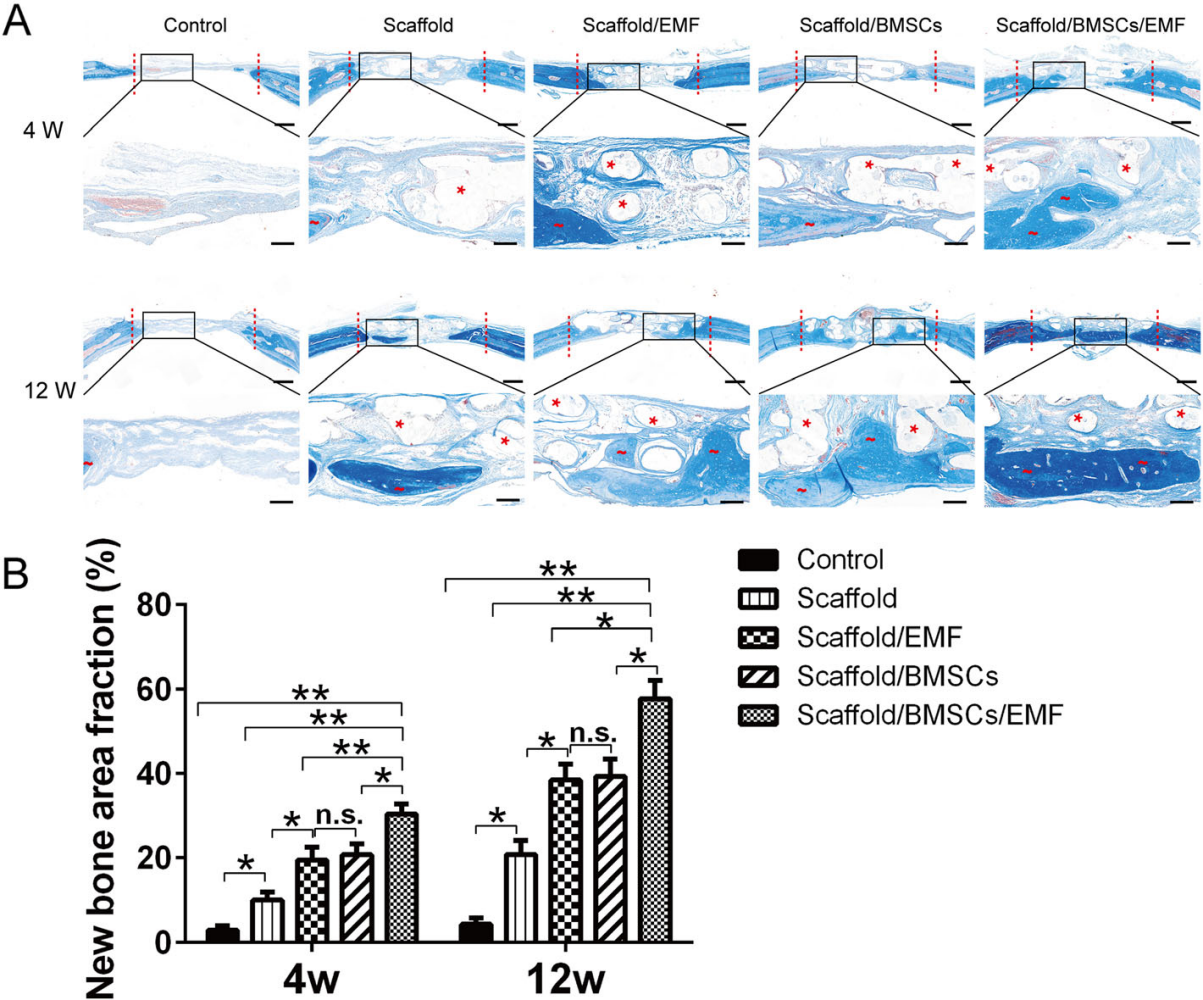
Bone regeneration evaluated by Masson’s trichrome staining. **a** Masson’s trichrome staining was conducted to show the newly formed bone and collagen deposition within the implanted constructs at 4 and 12 weeks post-surgery. The dotted line indicates the boundary of the 6-mm defect. Red wary lines designate newly formed bone, and asterisks indicate residual scaffolds. Scale bars in lower magnification images represent 1000 μm, and scale bars in higher magnification images represent 250 μm. **b** Quantification analysis of new bone area fraction in different groups at 4 and 12 weeks (*n* = 6). n.s. indicated no significance (^*^P < 0.05, ^**^P < 0.01)

New bone formation in the bone defect of different groups was assessed with HE staining 4 and 12 weeks after implantation (Fig. 9). Fibrous connective tissues with little newly formed bone were observed in control group 4 and 12 weeks post-operation. Compared to the other 4 groups, Scaffold/BMSCs/EMF group exhibited a better bone regeneration. Furthermore, the newly formed bone almost covered the calvarial defect in Scaffold/BMSCs/EMF group at 12 weeks. Masson’s trichrome staining was also employed to evaluate the bone regeneration (Fig. 10a), and same tendency was observed. Quantitative analysis of new bone area fraction of each group at 4 weeks and 12 weeks was further performed using Image-J software. As shown in Fig. 10b, Scaffold/BMSCs/EMF group showed the highest value among the five groups at each time set.

### Biomechanical evaluation of the craniums

**Fig. 11:**
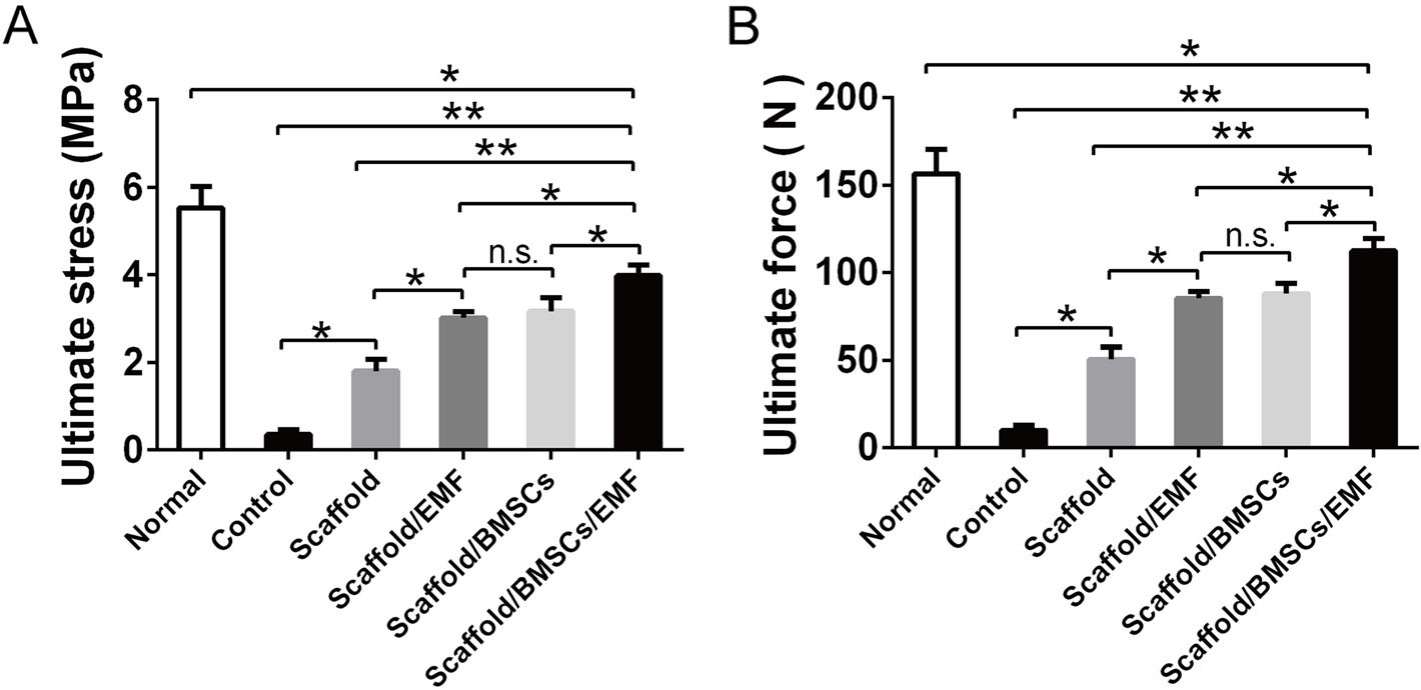
Biomechanical analysis of the craniums. **a** Ultimate stress and **b** ultimate force quantification analysis of the craniums in different groups at 12 weeks (*n* = 6). n.s. indicated no significance (^*^P < 0.05, ^**^P < 0.01)

Twelve weeks post-operation, a push-out test was used to evaluate the biomechanical properties of the craniums in each group. Results indicated that craniums of the scaffold/BMSCs/EMF group owned significantly greater ultimate stress and ultimate force compared to the other 4 groups (Fig. 11a, b).

## Discussion

Craniofacial reconstruction remains a great challenge in regenerative medicine. Compared with traditional regeneration methods, 3D-printing technologies offered implanted grafts with precise size and macroporous structure, which is essential for cell attachment, local vascularization, and bone ingrowth [30, 31]. Over the decades, incorporation of growth factors into grafts has been widely explored in bone tissue engineering [32]. However, high cost, short half-life, and limited production hinder this application [5]. Electromagnetic fields (EMF), as a safe, noninvasive strategy, may serve as a substitute of the former. However, standard EMF therapy remained uncertain due to controversial results [33]. EMF with different parameters including intensity, frequency, and duration varies in effects. We previously found sinusoidal EMF (15 HZ, 1 mT, 4 h/day) exhibited prominent immediate as well as lasting effects on bone regeneration [17, 25]. Therefore, we chose this parameter in this study. Unlike noncritical defect, critical-sized defect (CSD) is unable to be repaired spontaneously. Planned intervention is necessary in this animal model [23]. Based on CSD model, we only need to focus on the effects of determined strategy. Accordingly, we tried to combine specific magnetic therapy and 3D-printing for craniofacial reconstruction in this study, we expect to explore new avenue for bone regeneration.

Polylactic acid (PLA) and hydroxyapatite (HA) are common materials for 3D-printing. Both of them have their own advantages and limitations. Combining them into one composite material could minimize the drawbacks and maintain the advantages [34]. Moreover, PLA/HA composite scaffolds with a ratio of 4:1 were proved to present the optimal property [16]. Accordingly, we have fabricated the 3D porous scaffolds using a nozzle-deposition system. In the meantime, rat bone marrow mesenchymal stem cells (BMSCs) were isolated, identified and chosen as the seeded cells in our study. We firstly confirmed the physical properties and cyto-compatibility in vitro. Results showed the scaffolds showed high porosity (~ 70%) and suitable mechanical performance (compression strength of 31.18 ± 4.86 MPa and modulus elasticity of 10.12 ± 1.24 GPa). Furthermore, uniform macroporous structure and high seeding efficacy was observed under SEM. It is interesting to see the random small pores on the surface of the scaffolds at high magnification. These small pores may further contribute to the cell attachment, local vascularization and nutrition exchange. CCK-8 assay and LIVE/DEAD staining indicated that cells proliferated well on the scaffolds.

Generally, the proliferation and differentiation capacities are vital to seeded cells [35]. Researchers have been working on improving these two potentials in different approaches. Rashad et al. improved the proliferation of seeded MSCs via coating the scaffold with cellulose nanofibrils material [36]. Tiffany et al. promoted the osteogenic differentiation of seeded porcine dipose derived stem cells by fabricating a zinc functionalized scaffold [37]. In our study, CCK-8 kit and western blotting were performed to detect the effects of EMF on the proliferation and osteogenic differentiation of BMSCs cultured on scaffolds. Results proved EMF enhanced early cell proliferation (day 7 and day 9). Moreover, expression of osteogenesis-related protein (RUNX2 and OPN) was elevated with a 7 days EMF exposure. Previous study indicated that MAPK pathway was involved broadly in the cell proliferation and differentiation [38]. Meanwhile, EMF with various parameters was reported to play an important role in regulating cell proliferation and differentiation [39, 40]. Therefore, we continued to explore the activation of MAPK pathway. We believe this would further contribute to the understandings of the mechanism involved in the biological process of EMF therapy.

To evaluate the potential application of 3D-printed PLA/HA composite scaffolds with EMF therapy, a 6-mm rat critical-sized calvarial defect model was created. Micro-CT analysis demonstrated that Scaffold/BMSCs/EMF group showed the highest amount of bone formation within the defect region at 4 and 12 weeks. It is highly interesting to see that the scaffold/BMSCs/EMF group also showed a higher formation of new blood vessels within the defect at 6 weeks. There is no conclusive evidence indicating that EMF could promote vascularization in bone tissue engineering. It can be assumed that transplantation of BMSC-laden PLA/HA composite scaffolds with EMF exposure had stimulatory effects on local vascularization. Besides, this may attribute to the increased BMSCs on scaffolds stimulated by EMF post-operation. Previously study confirmed that undifferentiated BMSCs could commit to endotheliocyte [21], thus facilitating neovascularization of the engineered bone grafts. Moreover, histology analysis including HE and Masson’s trichrome staining further revealed that the scaffold/BMSCs/EMF group showed the best bone integration. As the mechanical properties play an important role in assessing the implanted constructs in tissue engineering [41], we also performed the push-out test in different groups at 12 weeks. Results suggested implants in scaffold/BMSCs/EMF group displayed the optimal biomechanical properties, despite not reaching the strength of the normal bone.

Since sinusoidal EMF with different parameters has diverse effects. Introducing magnetic therapy with multiple parameters into the study of bone tissue engineering would be more interesting. Furthermore, as the incorporation of various growth factors into regenerative medicine has gained huge attention over the last decades, it would be meaningful to compare the bone repair effect using EMF exposure vs. growth factors.

## Conclusions

**Fig. 12:**
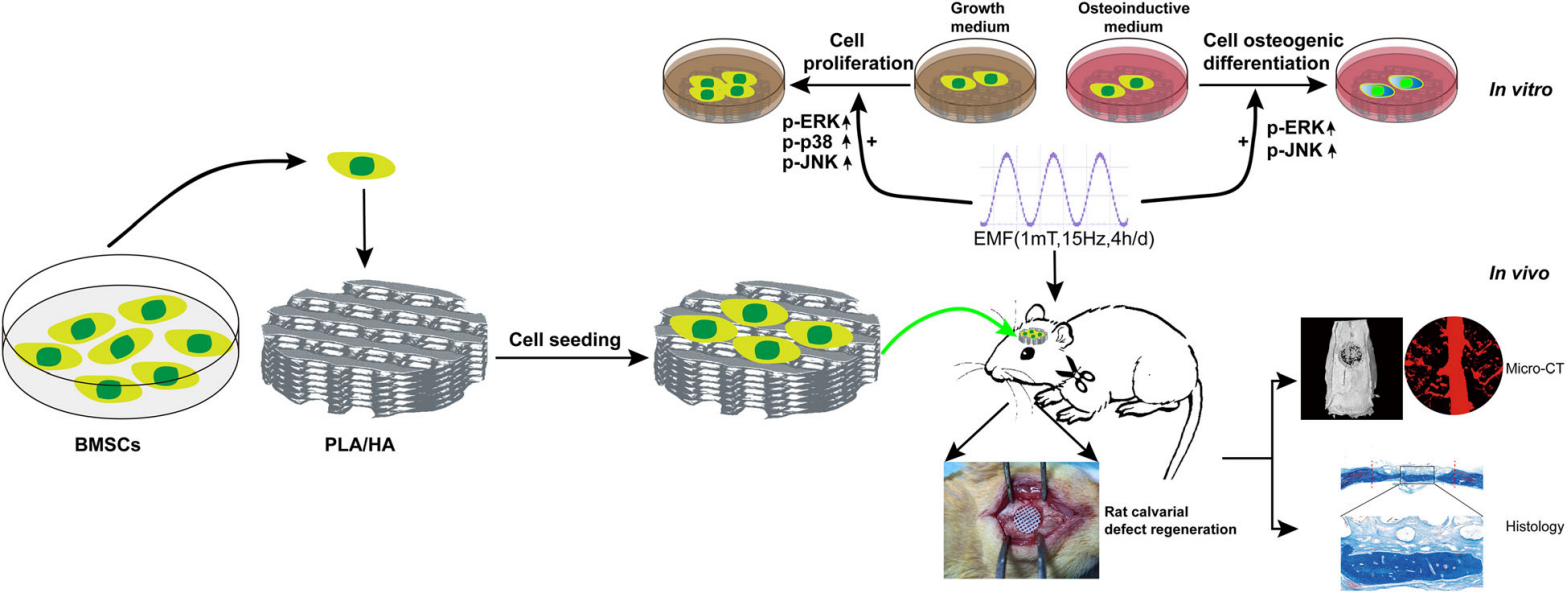
Schematic diagram of the effect of EMF on the BMSC-laden PLA/HA implants in vitro and in vivo. EMF exposure enhanced the proliferation and osteogenic differentiation potential of BMSCs cultured on PLA/HA composite scaffold by activating MAPK or MAPK-associated ERK and JNK pathways. Furthermore, BMSC-laden PLA/HA implants achieved great potentials in rat critical-sized calvarial defect regeneration

All above, we have successfully fabricated the 3D-printed composite scaffolds. The scaffolds showed macroporous structure, good mechanical properties, and excellent cyto-compatibility. Based on our previous study, we are the first to adopt the sinusoidal EMF (15 HZ, 1 mT, 4 h/day) in the reconstruction of critical-sized calvarial defect with 3D-printed technology. Our in vitro results proved that EMF promoted the proliferation and osteogenic differentiation of the BMSCs cultured on scaffolds and functioned partly by activating the MAPK or MAPK-associated ERK and JNK pathways. It is interesting to see that MAPK-associated p38 pathway was only activated in the process of EMF-induced BMSCs proliferation. The evaluation of the rat critical-sized calvarial defect models further demonstrated that implanted constructs showed a higher new bone formation and vascularization with EMF exposure. Furthermore, the biomechanical analysis displayed the same tendency (Fig. 12). Collectively, EMF treatment with 3D printing technologies might be a promising candidate for craniofacial reconstruction.

## Data Availability

The datasets used and/or analyzed during the current study are available from the corresponding author on reasonable request.

## Abbreviations

PLA: Polylactic
HA: Hydroxyapatite
BMSCs: Bone marrow mesenchymal stem cells
EMF: Electromagnetic fields
MAPK: Mitogen-activated protein kinase
CSD: Critical-sized defect
DMEM/F12: Dulbecco’s modified Eagle’s medium F12
OIM: Osteoinductive medium
CIM: Chondroinductive medium
AIM: Adipoinductive medium
GM: Growth medium
SD: Sprague-Dawley
BV: Bone volume
TV: Total volume
BMD: Bone mineral density
HE: Hematoxylin and eosin
